# Outside-host phage therapy as a biological control against environmental infectious diseases

**DOI:** 10.1186/s12976-018-0079-8

**Published:** 2018-06-08

**Authors:** Ilona Merikanto, Jouni T. Laakso, Veijo Kaitala

**Affiliations:** 1 Faculty of Biological and Environmental Sciences, University of Helsinki, Helsinki, Finland; 2 Department of Psychology and logopedics, Faculty of Medicine, University of Helsinki, Helsinki, Finland; 3 National Institute for Health and Welfare, Helsinki, Finland; 4 Centre of Excellence in Biological Interactions, University of Jyväskylä, Jyväskylä, Finland

**Keywords:** Bacteriophage, Columnaris disease, Environmental opportunist, *Flavobacterium*, Host-parasite interaction, SI model

## Abstract

**Background:**

Environmentally growing pathogens present an increasing threat for human health, wildlife and food production. Treating the hosts with antibiotics or parasitic bacteriophages fail to eliminate diseases that grow also in the outside-host environment. However, bacteriophages could be utilized to suppress the pathogen population sizes in the outside-host environment in order to prevent disease outbreaks. Here, we introduce a novel epidemiological model to assess how the phage infections of the bacterial pathogens affect epidemiological dynamics of the environmentally growing pathogens. We assess whether the phage therapy in the outside-host environment could be utilized as a biological control method against these diseases. We also consider how phage-resistant competitors affect the outcome, a common problem in phage therapy. The models give predictions for the scenarios where the outside-host phage therapy will work and where it will fail to control the disease. Parameterization of the model is based on the fish columnaris disease that causes significant economic losses to aquaculture worldwide. However, the model is also suitable for other environmentally growing bacterial diseases.

**Results:**

Transmission rates of the phage determine the success of infectious disease control, with high-transmission phage enabling the recovery of the host population that would in the absence of the phage go asymptotically extinct due to the disease. In the presence of outside-host bacterial competition between the pathogen and phage-resistant strain, the trade-off between the pathogen infectivity and the phage resistance determines phage therapy outcome from stable coexistence to local host extinction.

**Conclusions:**

We propose that the success of phage therapy strongly depends on the underlying biology, such as the strength of trade-off between the pathogen infectivity and the phage-resistance, as well as on the rate that the phages infect the bacteria. Our results indicate that phage therapy can fail if there are phage-resistant bacteria and the trade-off between pathogen infectivity and phage resistance does not completely inhibit the pathogen infectivity. Also, the rate that the phages infect the bacteria should be sufficiently high for phage-therapy to succeed.

**Electronic supplementary material:**

The online version of this article (10.1186/s12976-018-0079-8) contains supplementary material, which is available to authorized users.

## Background

Attempts to eliminate infectious diseases with antibiotics are in many cases challenging, especially concerning pathogens that are not solely dependent on the host for growth but also replicate independently of the host in the outside-host environment e.g. as saprotrophs. These pathogens can be referred to as environmentally growing opportunists [1–4]. Environmentally growing opportunist pathogens are abundant and cause increasing economical and health issues among both humans and cultivated species. Pathogens that fall under this category include for instance *Vibrio cholera*, *Pseudomonas aeruginosa, Legionella pneumophila, Listeria monocytogenes, Cryptococcus neoformans* and many species from genus *Mycobacterium, Flavobacterium* and *Serratia* [1, 3, 5–15].

Antibiotics may fail to eradicate the entire pathogen population even though all the infected hosts would be successfully treated, as the pathogen population is capable of surviving and growing independently of the hosts in the environment [4]. New disease outbreaks can thus occur even at a relatively short time period after conducting antibiotic treatments, as has been seen in the cases of cholera in humans [10] and the columnaris disease in cultivated freshwater fishes [13, 16, 17]. Attempts to vaccinate the host also may be problematic because immune allocation against a hypothetical pathogen may render host vulnerable to other pathogens or parasites [18]. Therefore, targeting solely the pathogen population potentially growing inside the hosts may not be a successful solution to disease control in the long run. Regarding fish diseases the antibiotic treatments have been challenging as the treatment is usually conducted by administering antibiotics in food mixtures and in many cases, such as in columnaris disease, the infected fish cease feeding [17, 19]. This increases the antibiotic residues that easily spread from the cultivation facilities to the environment from the water circulation system in fisheries and facilitate the evolution of multi-drug resistant bacterial strains [19]. Extensive use of antibiotics has promoted the spread of antibiotic resistance among the pathogenic bacteria. Furthermore, leakages of antibiotics to the environment cause disruption of the environmental microbial communities [19–21]. Disruption of the microbial communities can decrease microbial competition and therefore promote invasions of novel diseases and disease outbreaks [22, 23].

Phage therapy by bacteriophages has been considered as an alternative method to antibiotics in controlling infectious bacterial diseases [24]. Here, the interest is in therapies using lytic bacteriophages that infect the bacteria cell and break them down by lysis [25]. In many cases, including the columnaris disease, the phages are rather strain-specific and thus, contrary to antibiotics, they target primarily the intended bacterial disease agent and do not harm other bacteria strains [19, 26]. Phage therapy experiments have given both promising outcomes as well as failed to eliminate the disease. Experimental in vivo treatment of catfish infected by columnaris disease agent *F. columnare* by oral introduction of phages has been successful [27]. However, phage therapy has mostly considered the treatment of the hosts in vivo and not targeting the pathogen population in the outside-host environment [19, 28]. Another concern in phage therapy is the existing or rapid development of resistance against the bacteriophages [19, 24]. Methods for local elimination of the environmentally growing opportunist disease by targeting the pathogen population in the outside-host environment are needed to successfully prevent disease outbreaks. The idea of using phages to suppress the pathogen population in the outside-host environment for disease prevention was introduced by Levin and Bull already a decade ago [24]. They also raised the issue of phage-resistance and introduced preliminary phage-therapy models [24]. There are few previous phage-therapy models targeting environmental bacterial population [29, 30]. However, these models do not consider the phage-resistant competitors. Thus, phage-therapy theory omitting the possibility for phage-resistance is not realistic in long-term biological control [19].

Here, we study phage therapy as a biological control method against the environmentally growing infectious diseases by targeting the outside-host pathogen population. Parameterization of the model is based on the columnaris disease caused by saprotrophic bacterium *Flavobacterium columnare*, but the model can also be applied to other environmentally growing opportunist diseases. As columnaris disease causes severe infections in fresh-water fisheries worldwide, killing often all the fish in the infected tank in a matter of days [13, 17, 31], we chose pathogen-host parameters that would cause the fast death of the whole susceptible host population in the absence of the phage. We first analyze how introducing the phages into the host-pathogen system could eliminate the disease and how good at infecting the bacteria the phage should be in order to accomplish this. Secondly, we address the issue of resistance towards the phages by considering different trade-off scenarios between the pathogen infectivity and the resistance towards phages. We consider a scenario, where resistance towards the phages is associated with complete or partial loss of the pathogen infectivity.

## Methods

### The general model of phage therapy

We consider a deterministic continuous time model combining the environmentally growing opportunist bacterial pathogen-host interaction and the outside-host pathogen-bacteriophage interaction. The model combines SI dynamics based on model by Anderson and May (1981) [32] with the pathogen outside-host growth model [4] and the lytic viral infection by the bacteriophages to describe changes in time (*t*) in the densities of the susceptible hosts (*S*), the infected hosts (*I*), the pathogens (*P*), the phage-resistant bacteria (*B*) and the pathogen bacteriophages (*F*) in the environment outside-host.

The complete model of phage therapy is given as follows:

1 $$ \frac{dS}{dt}={r}_S\left(1-S\right)S-\beta SP-{\beta}_B SB-{\mu}_{SI}S+\delta I+\delta {I}_B $$

2 $$ \frac{dI}{dt}=\beta SP-\left(\alpha +{\mu}_{SI}\right)I-\delta I $$

3 $$ \frac{d{I}_B}{dt}={\beta}_B SB-\left(\alpha +{\mu}_{SI}\right){I}_B-\delta {I}_B $$

4 $$ \frac{dP}{dt}=\varLambda \alpha I+{r}_P\left[1-\left(P+B\right)/K\right]P-{\mu}_PP-{\beta}_F PF $$

5 $$ \frac{dB}{dt}={\varLambda}_B\alpha {I}_B+{r}_B\left[1-\left(P+B\right)/K\right]B-{\mu}_BB $$

6 $$ \frac{dF}{dt}={\varLambda}_F{\beta}_F PF-{\mu}_FF $$

The logistic density-dependent growth of the susceptible host (*S*, eq. 1) is determined by the growth rate *r*_*S*_ and the host carrying capacity, which is scaled to 1. Susceptible host population die at rate *μ*_*SI*_. This death rate represents mortality of the host through, e.g. predation, which is the main proximate cause of mortality in fishes in nature [33], or due to removal from the environment by harvesting. The susceptible host population is also suppressed as they are infected through environmental transmission of the pathogen at rates *β* and *β*_*B*_. The susceptible hosts can recover from the disease at rate *δ*.

The infected host population size (*I*, eq. 2) increases through environmental transmission rate *β* of the pathogens to the susceptible hosts depending on population sizes of *S* and *P.* We omitted direct transmission of the disease between hosts as columnaris disease is mainly transmitted via the environment and direct transmission of the disease is considered to be rare. We assume no resource competition between susceptible and infected hosts as diseased animals generally cease feeding, which is also the case regarding the fish infected by columnaris disease [17, 19]. Furthermore, the infected hosts are not able to reproduce once they have become infected and cease feeding, as is the case with columnaris infections. [17]. The infected hosts die due to the same causes as the susceptible hosts (*μ*_*SI*_) or due to the infection at a rate α (indicating virulence). The infected hosts recover from the disease at rate *δ*.

We also consider the situation where the phage-resistant bacteria are also able to transfer to the susceptible hosts at rate *β*_*B*_, which determines the increase of the phage-resistant infected host population size (*I*_*B*_*,* eq. 3) depending on population sizes of *S* and *B.* In these situations, the phage-resistant infected hosts die due to the disease (α) or due to the same causes as the susceptible hosts (*μ*_*SI*_).

The pathogen population in the outside-host environment (*P*, eq. 4) increases through the growth outside and inside the hosts. The infected hosts that die due to the disease at rate α and release new pathogens at rate *Λ*. The pathogen release rate *Λ* reflects the overall growth of the pathogen on the host through single infection independent of the infection time, as these pathogens are able to grow in a dead host; in columnaris disease, the release rate from the living hosts is minor as compared to release rate from the dead hosts [13]. Novel pathogens are only released from the infected hosts as they die to the infection and not when they die of other reasons (*μ*_*SI*_), indicating here mortality of the host due to predation or harvesting because this would be a dead-end for an ectoparasitic *F. columnare* [31]. The outside-host growth of the pathogen in eq. 4 is density-dependent, determined by the maximum growth rate *r*_*P*_ and the constant parameter *K* modifying the strength of density-dependence of the environmental growth rate. The pathogen *P* and the phage-resistant non-pathogenic bacteria *B* compete for the same resources in the environment*.* The pathogen population dies at rate *μ*_*P*_, describing e.g. protozoa predation, and due to infection by the bacteriophages at rate *β*_*F*_, which also describes the transmission rate of the bacteriophage to pathogenic bacteria. For simplicity, we assume that the lysis of the infected bacteria cells is instantaneous.

The phage-resistant bacteria population (*B*, eq. 5) increases through the density dependent saprotrophic growth in the environment with the growth rate *r*_*B,*_ which is influenced by the parameter *K* modifying the strength of density-dependence of the environmental growth rate*.* We omitted the mutation from pathogen to phage-resistant from the model as trivial because selection sustains a steady phenotypic heterogeneity of microbes in the environment and thus the availability of these resistant strains in the environment can be assumed [34–36].

Gaining resistance to the bacteriophages can result in a total loss of the pathogen infectivity, as has been seen in highly virulent *F. columnare* strains B67, B185 and B245 [37]. However, phage resistance in *F. columnare* does not necessarily mean a total loss of infectivity, even though there is a trade-off between the pathogen infectivity and the phage resistance. For instance, *F. columnare* strain (Os06) has been observed to cause infections at a low level even after development of phage resistance [37]. In the model, we thus allow in some scenarios the phage-resistant bacteria to increase inside the host at the rate *Λ*_*B*_, when the infected hosts die at rate α. In the analysis of the model we also consider an option where phage resistance results in a complete loss of the pathogen infectivity. The phage-resistant bacteria die at rate *μ*_*B*_*.*

The phage infection (*F*, eq. 6) leads to immediate lysis of the bacterial cell and production of phages, which are released from the infected pathogen cells at rate *Λ*_*F*_, and depending on the transmission rate *β*_*F*_. The phage population size decay at rate *μ*_*F*_*.*

### Parameterization of the model

The parameter values used in the stability analyses were selected to represent a large range of plausible biological values for environmentally growing opportunist pathogens and their potential hosts, especially regarding the columnaris disease. The parameter values used are given in Table 1. The parameter values were chosen to present a disease dynamics, where the pathogen drives the host towards extinction in the absence of viral infection by the bacteriophages (Additional file 1: Figure S1).

**Table 1:** Parameter values used in the analyses

Parameter	Explanation of the parameter	Parameter values	
α	Virulence (Mortality of the infected hosts due to infection)	0.1 (day^−1^)	in all the analysis
*β*	Pathogen transmission rate to susceptible hosts from environment	10^−5^ (day^−1^)	in all the analysis
*Λ*	Pathogen release rate from infected hosts when they die	10^9^	in all the analysis
*β*_*B*_	Phage-resistant pathogen transmission rate to susceptible hosts from environment	0–10^−10^ (day^− 1^)	in Fig. 3
		0 (day^− 1^)	in Figs. 1, 2; Additional file 1: Figure S1; Additional file 3: Figure S2 and Additional file 4: Figure S3
*Λ*_*B*_	Phage-resistant pathogen release rate from infected hosts when they die	10^5^	in Fig. 3
		0	in Figs. 1, 2; Additional file 1: Figure S1; Additional file 3: Figure S2 and Additional file 4: Figure S3
*β*_*F*_	Phage transmission rate to pathogens from the environment	10^−8^–10^− 4^ (day^− 1^)	in Fig. 1
		10^− 7^ (day^− 1^)	in Fig. 2
		10^− 3^ (day^− 1^)	in Fig. 3 and Additional file 4: Figure S3
		0	in Additional file 1: Figure S1
		10^− 9^–10^− 5^ (day^− 1^)	in Additional file 3: Figure S2
*Λ*_*F*_	Phage burst size from infected pathogen	100	in Figs. 1, 2 and 3 and Additional file 4: Figure S3
		0	in Additional file 1: Figure S1
		0.0001–10	in Additional file 3: Figure S2
*r*_*S*_	Susceptible host growth rate	0.1 (day^− 1^)	in all the analysis
*r*_*P*_	Pathogen growth rate outside-host	3 (day^−1^)	in all the analysis
*r*_*B*_	Phage-resistant bacteria growth rate outside-host	3 (day^−1^)	in Fig. 3
		0 (day^− 1^)	in Figs. 1, 2; Additional file 1: Figure S1; Additional file 3: Figure S2 and Additional file 4: Figure S3
*K*	Constant parameter K modifying the strength of density-dependence of the environmental growth rate	10^9^	in all the analysis
*μ*_*SI*_	Mortality of the susceptible and infected hosts due to other reasons than infection	0.001 (day^−1^)	in all the analysis
*μ*_*P*_	Pathogen mortality outside-host	0.1 (day^−1^)	in all the analysis
*μ*_*B*_	Phage-resistant bacteria mortality outside-host	0.1 (day^−1^)	in Fig. 3
		0 (day^− 1^)	in Figs. 1, 2; Additional file 1: Figure S1; Additional file 3: Figure S2 and Additional file 4: Figure S3
*μ*_*F*_	Phage decay rate outside-host	0.1 (day^− 1^)	in Figs. 1, 2, 3; Additional file 3: Figure S2 and Additional file 4: Figure S3
		0 (day^− 1^)	in Additional file 1: Figure S1
*δ*	Recovery of the hosts from infection	0 (day^− 1^)	Figs. 1, 3; Additional file 1: Figure S1; Additional file 3: Figure S2 and Additional file 4: Figure S3
		0–0.8 (day^− 1^)	in Fig. 2

The growth rate of *S* (*r*_*S*_) corresponds the growth rate of the multicellular hosts of the environmentally growing opportunistic pathogen bacteria *F. columnare* [4]. The outside-host growth rate of *P* and *B* (*r*_*B*_ and *r*_*P*_) were set to correspond to the growth rate of the environmental bacteria and the environmentally growing pathogens, such as *F. columnare* and *S. marcescens* [4, 38–40]. The constant parameter *K* modifying the strength of density-dependence of the environmental growth rate was set to correspond the bacteria population densities seen in the environment [38]. The growth of the pathogens inside the host (*Λ* and *Λ*_*B*_, indicating release rate of new pathogens from infected hosts) was set to present realistic pathogen shedding rates from a fish infected with the columnaris disease [13]. The phage burst size from an infected pathogen cell (*Λ*_*F*_) corresponds the observed phage burst size per bacteria cell in experimental studies [41–43]. The burst size can however vary quite a lot, for instance in T4 bacteriophage burst size depends strongly on the age of the bacteria cell culture [43].

Infectivity of the pathogen (*β*, transmission rate to susceptible host) was set relatively low because in the environmentally growing opportunist pathogens the infectivity is generally assumed to be lower than in the obligate pathogens, with the exception of immunocompromised hosts [44].

The phage-resistant pathogen infectivity (*β*_*B*_, transmission rate to susceptible host) was varied on lower levels than *β*. This is due to the trade-off between the phage resistance and infectivity, and because the *F. columnare* strain is known remain phage-resistant while transforming back to the infective type has low infectivity [37]. The phage transmission rate to the pathogens (*β*_*F*_) was varied to explore how good at infecting the pathogens the phage needs to be to accomplish the elimination of the disease.

Virulence (*α*), indicating here the death rate of the infected hosts to the disease, was set to present realistic mortality to the infection by columnaris disease [4, 13, 17]. Here, we are considering a situation where the disease will cause the extinction of the hosts in the absence of disease control thus exceeding mortality due to e.g. harvesting or predation. This can be the case for instance when considering columnaris disease epidemics in fish farms, where the infection can kill the whole fish tank [13, 17]. This is why we have set the background mortality of the hosts (*μ*_*SI*_) lower than *α*. The pathogen and the phage-resistant bacteria mortality (*μ*_*P*_ and *μ*_*B*_) corresponds the observed death rate in the aquatic bacteria [45, 46]. The phage decay rate (*μ*_*F*_) corresponds to those reported in previous studies [47, 48]. Finally, we assume in our main analyses that there is no recovery from the infection as columnaris disease is often lethal [13]. We however analyze separately how the recovery of the hosts would affect the disease dynamics under the situation where the phage-resistant strains are non-pathogenic by varying it under realistic values.

### Analysis

We analyzed the long-term ecological dynamics of our phage-therapy models analytically and numerically. We focused on three major model assumptions: First, we assume that there are no phage-resistant competitors of the pathogen present. This analysis highlights how the presence of a bacteriophage infecting the bacteria at different rates affects the environmentally growing disease dynamics (*S-I-P*) by decreasing the pathogen population density. Second, we consider how effective the phage therapy is in the presence of phage-resistant competitors, when phage-resistance comes at the cost of losing the pathogen infectivity completely. Third, we decrease the strength of the trade-off between phage resistance and infectivity, i.e. by assuming that some degree of infectivity is possible among phage-resistant competitors. When the phage-resistant competitors were absent, the stability analysis of the positive equilibrium *S, I, P, F > 0* was carried out by varying the phage transmission rate (*β*_*F*_) and the phage burst size (*Λ*_*F*_) simultaneously.

Bifurcation analyses were obtained by simulating the corresponding differential equation model until the dynamics were stabilized, that is, the initial transient was removed. These attractors were presented in the figures for the stable equilibria or stable periodic solutions. Stable equilibria were also calculated analytically. The bifurcation parameter is presented on the x-axis. Local stability properties of the equilibria were studied by calculating numerically the eigenvalues of the corresponding Jacobian matrices (Additional file 2).

The disease dynamics were also analyzed numerically using non-equilibrium initial densities for the pathogen, the phage, the phage-resistant and the host populations. Simulation length of 350 days was sufficient to uncover the long-term dynamics. Bifurcation diagrams were obtained by scoring the minimum and maximum values of the population fluctuations after removing the initial transient phase. First, the phage transmission rate (*β*_*F*_) was varied in the absence of phage-resistant competitors in bifurcation diagrams, with 20 different evenly distributed values from the value range used. Second, the recovery rate of the hosts from the disease (*δ*) was varied in the absence of phage-resistant competitors. Third, the phage-resistant non-pathogen growth rate (*r*_*B*_) was varied. Finally, the phage-resistant transmission rate to the susceptible host (*β*_*B*_) was varied when phage resistance did not result in the total loss of the pathogen infectivity. The numerical simulations of the model were performed with MATLAB v. 2016a ODE15s solver (Mathworks).

## Results

In order to support a thorough analysis of the host-pathogen-phage system we begin by commenting two simple community models, namely two-bacteria strain-phage system (*P-B-F*) and host-two pathogen system (*S-P-B*).

### Epidemiological dynamics in the absence of the host (P-B-F)

The two bacteria strains compete for the resources they obtain from the environment. If the immunity to the phage of the bacteria strain *B* has developed without any effect on the growth rate or mortality then, in the absence of the phage, their coexistence dynamics would be equal resulting in neutrally stable equilibrium, where the equilibrium levels of the bacteria strains ($\overline{P},\overline{B}$) are not defined uniquely. Introducing a phage in this system will cause additional mortality to the bacteria strain *P* without resistance towards phages. Thus, in the presence of the phage, the bacteria strain *B* would gain a competitive advantage and the non-resistant bacteria strain *P* would be driven to extinction. On the other extreme, the immunity to the phage of the bacteria strain *B* develops with a full cost of losing the ability to grow in the environment (*r*_*B*_ = 0). In this case, the bacteria strain *B* would become extinct and the equilibrium levels of the non-resistant bacteria strain *P* and the phage would be $\overline{P}=\frac{\mu_F}{\varLambda_F{\beta}_F}>0$, and $\overline{F}=\frac{\left\{{r}_P\left[1-\frac{\overline{P}}{K}\right]-{\mu}_P\right\}}{\beta_F}>0$.

### Epidemiological dynamics in the absence of phage

We next consider the case where the two pathogens attack the host in the absence of the phage. We confine ourselves to a simplified but illustrative case where the pathogen *B* is inferior competitor as compared to the pathogen *P*. In particular, we refer to the case analyzed below where the competitive dynamics of the pathogens in the environment are equal but the pathogen *P* is more efficient in multiplying within the host than the pathogen *B* (*r*_*P*_ = *r*_*B*_, μ_P_ = μ_B_, *Λ*_*P*_ > *Λ*_*B*_ and *β*_*P*_ > *β*_*B*_. According the well-known competitive exclusion principle, pathogen *P* outcompetes pathogen *B* unless the host is driven into extinction. As a whole, the analysis of the competition of infective pathogens is challenging and complicated (for a more thorough analysis of the competitive pathogens, see references by Anttila et al. 2013 and Merikanto et al. 2014 [22, 23]).

### Epidemiological dynamics in the absence of phage-resistant competitor

We first consider the community *S-I-P*-*F*, where the competing pathogen is absent (*B* = 0). We note that due to the environmental growth capacity the pathogen is always able to invade the system when alone or when the host is present. Thus, the possible patterns of species coexistence are 1) the pathogen alone (*P* > 0, *S*=*I*=*F* = 0), if the pathogen drives the host population to extinct and the phage is not introduced into the system; 2) the pathogen and the host coexist (*P, S, I* > 0, *F* = 0), the phage is not introduced into the system; 3) the pathogen, the host and the phage coexist (*P, S, I, F* > 0); 4) the host alone, after the phage has eradicated the pathogen.

We next assume that the pathogen *P* eliminates the host in the absence of disease control (Additional file 1: Figure S1). In a community (*P, S, I*), the pathogen drives the host extinct if ${r}_S-{\mu}_{SI}-\beta \frac{\left[{r}_P-{\mu}_P\right]K}{r_P}<0$, or $\beta >\frac{\left({r}_S-{\mu}_{SI}\right){r}_P}{\left[{r}_P-{\mu}_P\right]K}$ (see the Additional file 3). After eliminating the host, the pathogen settles down on the level $\overline{P}=\left({r}_P-{\mu}_P\right)K/{r}_P$. Interestingly, the ability of the pathogen to drive the host extinct does not depend on the release rate. On the other hand, when the phage is present the host is able to recover when${r}_S-\beta \overline{P}-{\mu}_{SI}>0$, or $\overline{P}<\left({r}_S-{\mu}_{SI}\right)/\beta$(see the Appendix, S2). Recalling that $\overline{P}={\mu}_F/{\varLambda}_F{\beta}_F$, the condition becomes *β*_*F*_ > *βμ*_*F*_/(*r*_*S*_ − *μ*_*SI*_)*Λ*_*F*_.

**Fig. 1:**
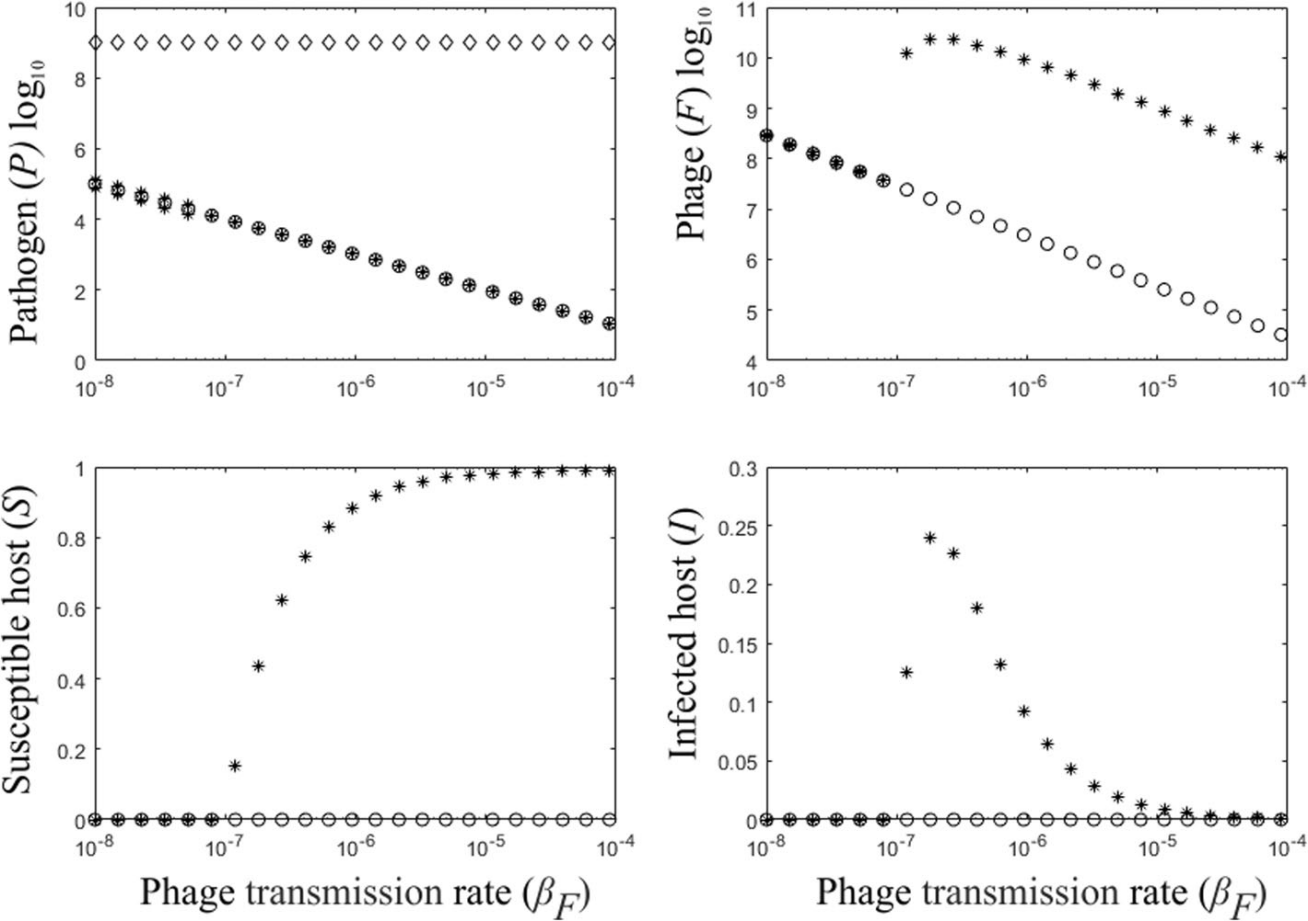
Bifurcation diagrams of the model dynamics in the absence of the phage-resistant bacteria population (*B* = 0), presenting equilibrium values of the susceptible host (*S*), the infected hosts (*I*), the pathogen (*P*) and the bacteriophage (*F*) population densities for the phage transmission rate, 10^− 8^<*β*_*F*_ < 10^− 4^. The x-axis is log10 scale. When the phage transmission rate is low (*β*_*F*_ < 10^− 7^), the susceptible and the infected hosts go asymptotically extinct while there is a stable coexistence of the pathogen and the phage. When the phage transmission rate increases, the pathogen population size decreases while the susceptible host population increases, close to their carrying capacity at higher phage transmission rates. The number of the infected hosts abruptly increases as phage transmission increases, but decrease asymptotically towards zero at higher phage transmission rates. The parameter values used are shown in Table 1. Pathogen level in the absence of the host and phage is indicated by “◊”. The coexistence equilibria of the pathogen and the phage in the absence of the host are denoted by “o”. The equilibrium solutions of all populations in the presence of the phage are denoted by “*”

We ask weather introducing the bacteriophages into the system allows the recovery of the host population. The effect of introducing a phage into the system can be divided into three areas of impact: 1) When the phage transmission rate is low (*β*_*F*_ < 10^− 7^) the phage is unable to rescue the host. The dynamics of the coexisting pathogen and phage are stable while the susceptible and infected hosts are absent due to the disease (Fig. 1). 2) When the phage transmission rate increases, the pathogen abundance decreases such that host population is able to survive. The susceptible host population size and the number of diseased individuals increase (Fig. 1). At the same time, the population size of the bacteriophage increases. 3) Finally, when the phage transmission rate continues to increase the disease begins to be under control and the susceptible host population size approaches its carrying capacity. The number of the infected hosts increases abruptly, after which their number decrease asymptotically towards zero (Fig. 1). Low levels of pathogen remain, keeping the phage present.

The previous analysis of the effect of the phage transmission rate can be repeated for the release rate *Λ*_*F*_ of the phage, as the effects are qualitatively similar. Local stability analysis shows that the positive equilibria for *S, I, P, F* > 0 exist and are locally stable when the phage transmission and the release rates are both high enough. On the other hand, the coexistence of all the populations is not possible when the phage transmission and the release rates are both low (*Λ*_*F*_ < 1 and *β*_*F*_ < 10^− 6^) or either of them is low (*β*_*F*_ close to 10^− 6^ or less, or *Λ*_*F*_ close to two or less). In these cases, the susceptible hosts will die out. The phage is unable to survive when both the phage transmission and the release rate are very low (*Λ*_*F*_ < 1 and *β*_*F*_ < 10^− 6^). The pathogen is able to remain in the system regardless of the absence of the host due to its ability to grow environmentally outside of the host (Additional file 3: Figure S2). When both the phage transmission and the release rates are high (*β*_*F*_ = 10^− 3^ and *Λ*_*F*_ = 100), the pathogen population decrease close to zero almost immediately and the decrease in the infected host population occurs with a delay (Additional file 4: Figure S3).

### Joint effect of the recovery and the phage treatment

**Fig. 2:**
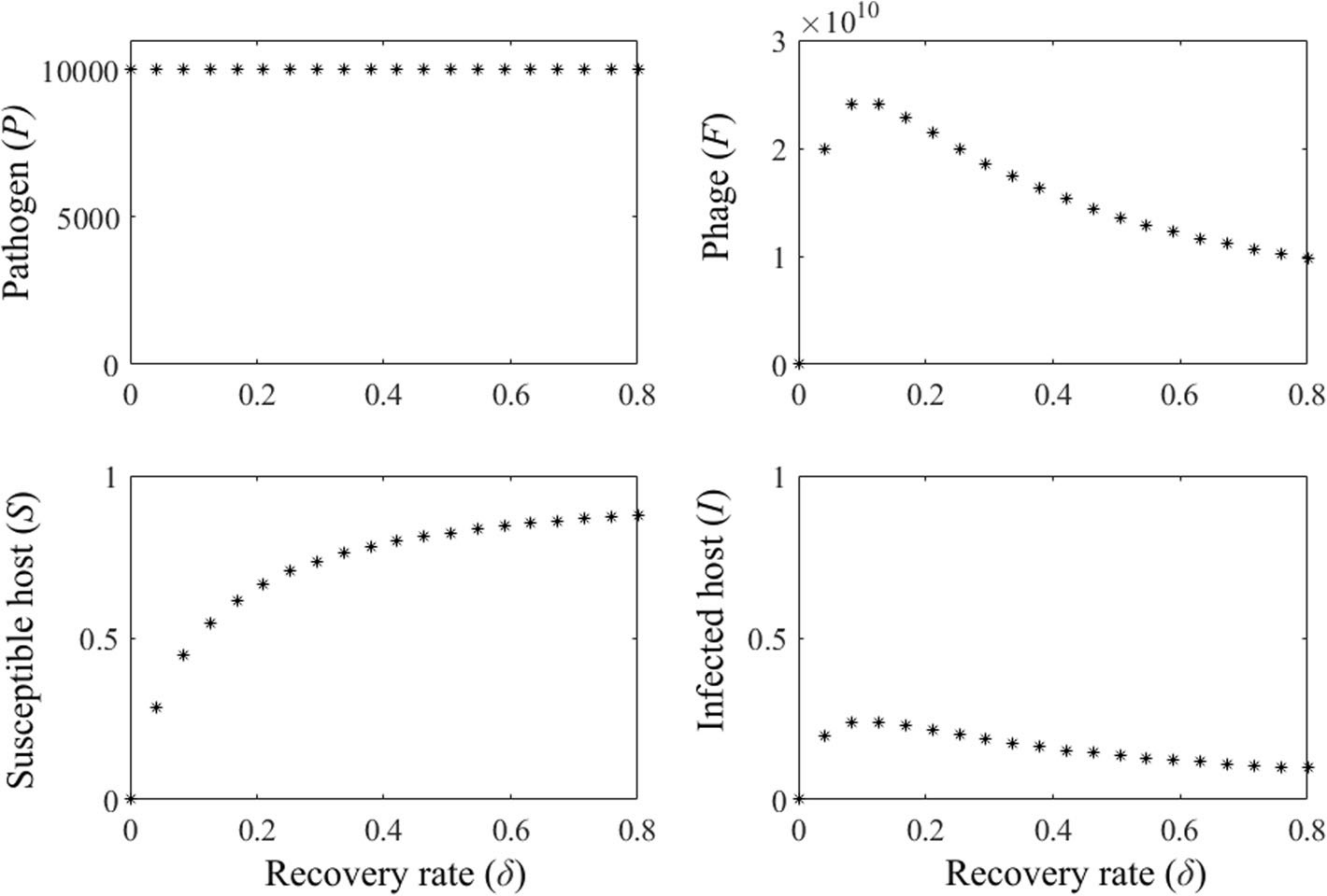
Bifurcation diagrams of the model dynamics in the absence of the phage-resistant bacteria population (*B* = 0), presenting population densities of the susceptible host (*S*), the infected hosts (*I*), the pathogen (*P*) and the bacteriophage (*F*) for the host recovery rate, 0 < *δ* < 0.8. When the recovery of hosts is not possible, the host are extinct. Increasing the recovery of hosts, increases the host and phage population sizes as well as the number of infections, while recovery rates above 0.2 decrease the number of infections and the population size of the phage. Pathogen populations remain stable regardless of the recovery rate of the hosts. The parameter values used are shown in Table 1

In the absence of the phage the pathogen stabilizes to its environmental carrying capacity (≈10^9^) after driving the host extinct, irrespective of the infected host recovery rate. Introducing the phage drives the pathogen population to low level (*P* = 10^4^). Increasing recovery rate *δ* increases the density of the susceptible hosts. The density of infected hosts is increased because the infection potential of the pathogen increases and more susceptible hosts are enter the infected population. Also, the density of the phages is increased due to the increased growth of the pathogen, which in turn allows increase of the phage population when there are more pathogens to infect by the phages. In more detail, low recovery rates (*δ* < 0.2) increase the densities of the infected hosts and phage, while the recovery rates above 0.2 decrease the densities of the infected hosts, as they are turning back as susceptible hosts faster, and phage, as lower amount of pathogens are released to the environment from infected hosts limiting phage growth potential. Pathogen population densities are not affected by the recovery rate of the hosts (Fig. 2). Thus, the recovery helps the host to survive when the phage is not extremely efficient.

### Epidemiology in the presence of phage -resistant non-pathogenic competitors

When a phage-resistant non-pathogenic competitor is introduced into the system it does not affect the disease dynamics or phage therapy. The phage drives the pathogen to a typical low level and the competitor then fills up the resource niche. In this case, the lower the pathogen level is, the higher the competitor level becomes. The competitor has no positive or negative role – it only utilizes the resources left by the pathogen. Moreover, these results are independent of the competitor’s growth rate *r*_*B*_.

### Epidemiology in the presence of competition with the less pathogenic phage-resistant form

**Fig. 3:**
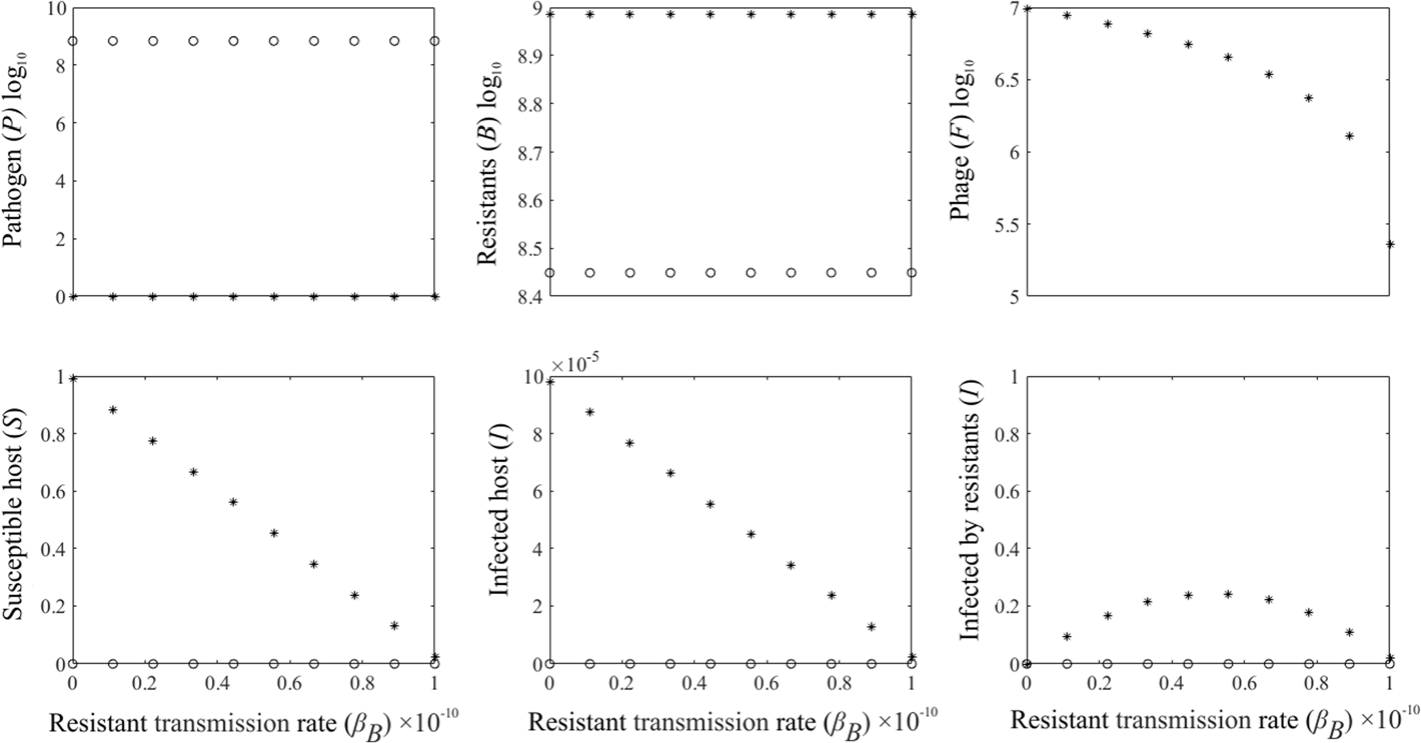
Bifurcation diagrams of the model dynamics with a competing pathogenic phage-resistant bacteria population. The panels represent the pathogen (*P*), the phage-resistant (*B*) the bacteriophage (*F*), susceptible host (*S*), the non-resistant infected hosts (*I*), the phage-resistant infected hosts (*I*_*B*_), population densities for the phage-resistant transmission rate to the susceptible hosts, 0 < *β*_*B*_ < 10^− 10^. The x-axis is log10 scale. When the phage is absent (o) the pathogens drive the host extinct. When the phage is introduced into the system (*) pathogen *P* is driven on a low level. Now, the abundance of the pathogen *B* drives the disease dynamics. With increasing infectivity the host population size deceases. The number of the diseases caused by pathogen *P* remains low due to the phage-control. The disease occurs still due to the increased abundance of the pathogen *B*. However, the increased infectivity on *β*_*B*_ ultimately drives the host population extinct. The parameter values used are shown in Table 1

Next we present a scenario, where the phage resistance of the competing pathogen is traded off with the ability to infect the susceptible hosts but does not fully remove infectivity. When the phage is absent then the less virulent bacteria can still kill the host. Neither of the pathogen types receives any advantage from infections since the host has now gone extinct, and consequently, the dynamics of the two pathogens are equal (*r*_*P*_ = *r*_*B*_ and *μ*_*P*_ = *μ*_*B*_). As a result, the coexistence dynamics of the two forms of the pathogens are now neutrally stable making their relative abundance arbitrary (Fig. 3).

When the phage is introduced into the system the pathogen *P* will be driven to a low level and a small proportion of the hosts are infected by this pathogen. Due to pathogen resistance against the phage and the reduced competition, the pathogen *B* will increase close the environmental carrying capacity. Increasing transmission rate reduces the susceptible population size. Also, increasing transmission rate first increases the number of infected hosts. Reduced susceptible population size makes the number of infected host to decrease again.

## Discussion

We have considered phage therapy targeting the outside-host population of environmentally growing pathogens. Our results show that phage therapy can in certain situations be efficient in eliminating the disease but may fail in others.

We first examined phage therapy in the absence of phage-resistant competitors of the pathogen. Here, the presence of a high-transmission phage successfully enable the recovery of the host population that would in the absence of the phage go asymptotically extinct due to the disease. Interestingly, an increase in the number of infections is seen as phage transmission increases, which results from improved growth potential of the susceptible hosts as the pathogen is diminished by the phage. Higher phage transmission rates decrease the number of infections by improved depletion of pathogens in the outside host environment. The rate of host recovery is faster when the phage transmission rate is high as the phage is able to deplete the infective pathogen population more successfully. However, if the phage has either low transmission or release rate the phage therapy is not successful in preventing local host extinction.

Few phage therapy models have been developed for treating the populations of cholera both inside and outside hosts [29, 30]. These models address phage-therapy similarly as we do in our first scenario, where we assume that there are no phage-resistant competitors present. Phage therapy models without the presence of phage-resistant strains are, however, often unrealistic in long-term treatment as the spread of the phage resistance among the bacteria is common and usually quite rapid [19].

Experimental studies have established that an extreme trade-off between the pathogen infectivity e and the phage resistance exists among virulent *F. columnare* strains that are the main pathogens causing severe columnaris infections [37]. For columnaris disease, the lack of pathogen virulence is comparable to the lack of infection ability as the pathogen is mainly released only as the host dies to the disease. We thus considered a situation where phage-resistant and non-resistant strains compete in the environment. If the phage-resistant competitor cannot cause infections, it does not affect the disease dynamics and only utilizes the outside host resources filling in the resource niche when the phage is able to keep the pathogen densities low.

The trade-off between the pathogen infectivity and the phage resistance is not always clear-cut such that it results in a complete loss of infectivity and death the hosts. The Os06 strain of *F. columnare* is for instance still able to cause infections at a low level even after developing phage resistance [37]. Thus, we also considered an option where the phage resistance does not fully inhibit the ability to infect the hosts even though the ability to infect the hosts is lower than among the pathogens without the phage resistance. Under these assumptions, where the phage resistance reduces the infectivity but does not completely inhibit the pathogen infectivity, stable coexistence of the pathogen, the phage-resistant pathogen, the phage, and the susceptible and infected hosts is possible when the phage-resistant transmission rate is low. Here, the pathogen population exists in a very low density. Once the phage-resistant transmission rate increases slightly the phage-resistant pathogens with low infectivity drive the host asymptotically extinct. When the hosts are extinct, the non-resistant pathogen and the phage-resistant pathogen persist in a stable coexistence in the outside-host environment. In this situation, the phage therapy fails and other methods for lowering the pathogen population sizes in the environment should be considered. For instance, controlling the disease by suppressing the pathogen populations outside-host by using protozoan predators could be a promising alternative [49].

Allowing hosts to recover from the disease affects the population densities of the susceptible and infected host, and phage, but not the pathogen. Low recovery rates of the host increase the population densities of susceptible host as well as infected host and phage. The increase in susceptible host population density as recovery is increased under the low value range enables better infection potential for the pathogen, which is seen as increased infections. This would increase the population density of pathogen without the presence of the phage. However, the phage population increases as pathogen growth is improved. Higher recovery rates of the host increase the population density of the susceptible host even further but decrease the population densities of the infected host and phage by reducing the pathogen population growth.

Our analysis shows that the success of phage-therapy in biological control of this disease class is highly variable, depending on the parameterization of the model. Thus, we argue that the knowledge of the biological system is essential in order to predict the outcome for the disease control by phage therapy in practice. It is also hypothetically possible that a more efficient phage in infecting pathogens could be inferior in competition with a less infective phage, when it comes to other traits, such as survival time in the outside-pathogen environment. Using phage-therapy in controlling diseases affecting for instance fish farms, continuous feed of phages to fish tanks during the epidemic periods might be necessary in assuring the success of phage-therapy.

It has been suggested that using phage cocktails with multiple species of phage rather than single phage species could be more efficient in lowering the bacterial densities, as has been shown in the laboratory with *V. cholerae* [50]. Using a single phage against the pathogen population might not be efficient as the phages are rather strain-specific and the pathogen population might consist of different pathogen strains, as is the case regarding columnaris disease in fish tanks [19, 26, 39]. The success of phage therapy however depends on which strains are present in the pathogen population.

## Conclusions

Our model analyses have demonstrated that phage therapy can fail if there are phage-resistant bacteria and the trade-off between pathogen infectivity and phage resistance does not completely inhibit the pathogen infectivity. Also, the transmission rate of the phages influences the success of phage therapy. Thus, it is critical to assess these factors to gain successful phage therapy in practice by conducting experiments to determine what is the infection ability and burs size of different phages when affecting specific pathogen strains in relation to a disease caused by an environmentally growing pathogen and how the pathogen virulence is changed in response to gaining phage resistance.

## Additional files

Additional file 1: **Figure S1** Time dynamics of pathogen, susceptible and infected host dynamics (*S-I-P*) in the absence of viral infection by bacteriophages and competition between the phage-resistant bacteria and the pathogen. X-axis and y-axis show time in days and the population density, respectively. Parameter values used are given in Table 1. In the absence of bacteriophages and phage-resistant bacteria, the pathogen (P), drives the susceptible host quickly extinct after which the infected host population goes extinct as well and the pathogen population stabilizes to grow saprothrophically in the absence of susceptible hosts. (TIF 2383 kb)

Additional file 2: Equilibrium population densities and the Jacobian matrices for phage therapy model (1)–(6). Local stability analysis of the P-S-I submodel at S=*I* = 0 in the absence of recovery. (DOCX 30 kb)

Additional file 3: **Figure S2** Local stability analysis for susceptible host, infected host, pathogen and bacteriophage dynamics (*S*-*I*-*P*-*F*) when the phage transmission and release rate are varied (10^− 9^ < *β*_*F*_ < 10^− 5^ and 0.0001 < *Λ*_*F*_ < 10). The positive equilibrium *S*, *I, P, F* > 0 is locally stable for high values of *Λ*_*F*_ and *β*_*F*_ (blue area). The coexistence equilibrium does not exist for lower values of when *Λ*_*F*_ and *β*_*F*_ (yellow area). In this case *S* and *I* become asymptotically extinct. Pathogen *P* always survives and even coexists with the phage *F* in the absence of the host *S*. The parameter values used are shown in Table 1. (TIF 2096 kb)

Additional file 4: **Figure S3** Time dynamics of pathogen, susceptible, infected host and bacteriophage dynamics (*S-I-P-F*) when the phage transmission and release rate are high (*β*_*F*_ = 10^− 3^ and *Λ*_*F*_ = 100). X-axis shows time in days and y-axis the population density. The pathogen population decreases close to extinction almost immediately, while there is a delay of approximately 47 days for the elimination of the disease and more than 50 days for decrease of the phage population close to extinction. The parameter values used are shown in Table 1. (TIF 2818 kb)
