# Supplementary figures and images for: Outside-host phage therapy as a biological control against environmental infectious diseases
Source: Theor Biol Med Model. 2018 Jun 8;15:7. doi: 10.1186/s12976-018-0079-8 (PMC5992827; doi:10.1186/s12976-018-0079-8)

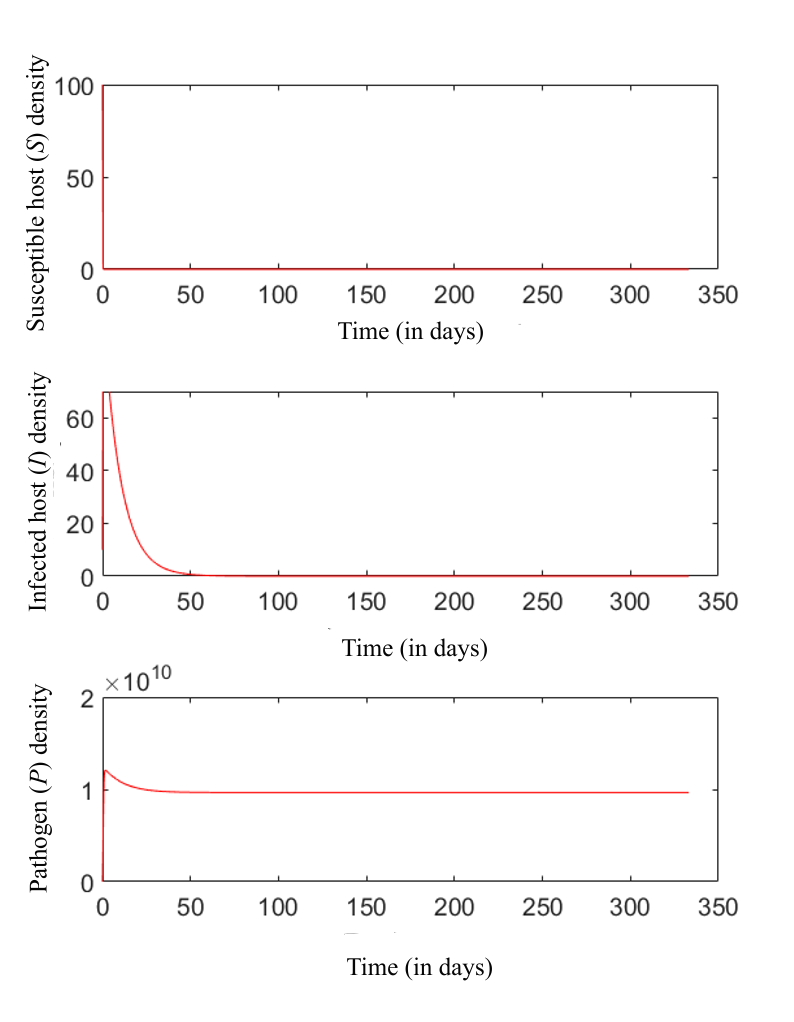

Supplement: Supplementary file 1 — Figure S1 Time dynamics of pathogen, susceptible and infected host dynamics (S-I-P) in the absence of viral infection by bacteriophages and competition between the phage-resistant bacteria and the pathogen. X-axis and y-axis show time in days and the population density, respectively. Parameter values used are given in Table 1. In the absence of bacteriophages and phage-resistant bacteria, the pathogen (P), drives the susceptible host quickly extinct after which the infected host population goes extinct as well and the pathogen population stabilizes to grow saprothrophically in the absence of susceptible hosts. (TIF 2383 kb) [file 12976_2018_79_MOESM1_ESM.tif]

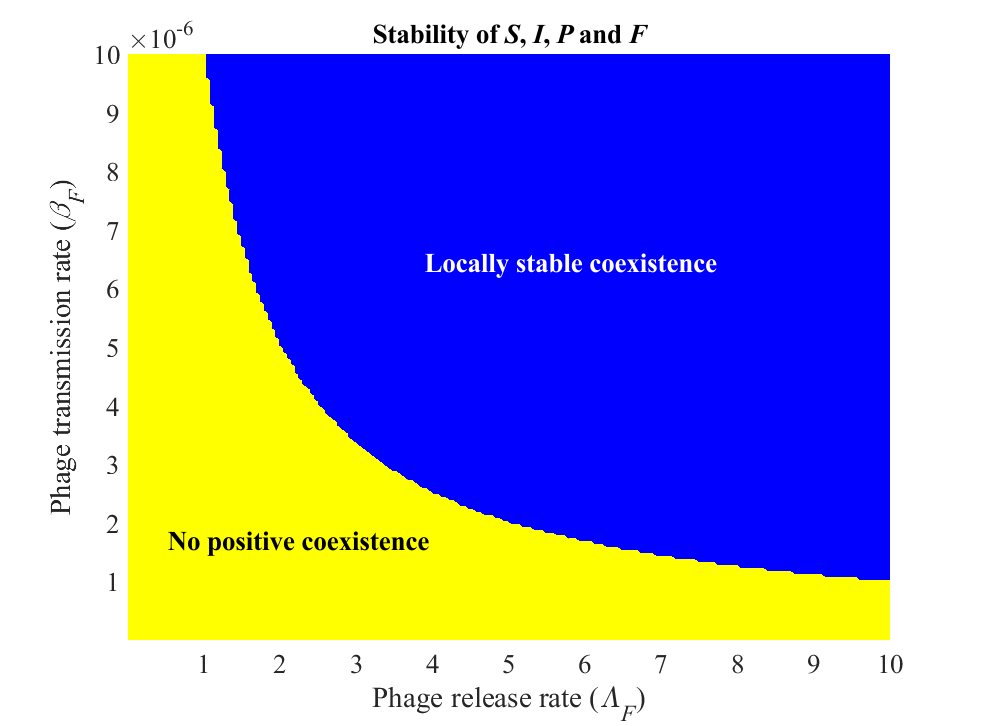

Supplement: Supplementary file 3 — Figure S2 Local stability analysis for susceptible host, infected host, pathogen and bacteriophage dynamics (S-I-P-F) when the phage transmission and release rate are varied (10− 9 < βF < 10− 5 and 0.0001 < ΛF < 10). The positive equilibrium S, I, P, F > 0 is locally stable for high values of ΛF and βF (blue area). The coexistence equilibrium does not exist for lower values of when ΛF and βF (yellow area). In this case S and I become asymptotically extinct. Pathogen P always survives and even coexists with the phage F in the absence of the host S. The parameter values used are shown in Table 1. (TIF 2096 kb) [file 12976_2018_79_MOESM3_ESM.tif]

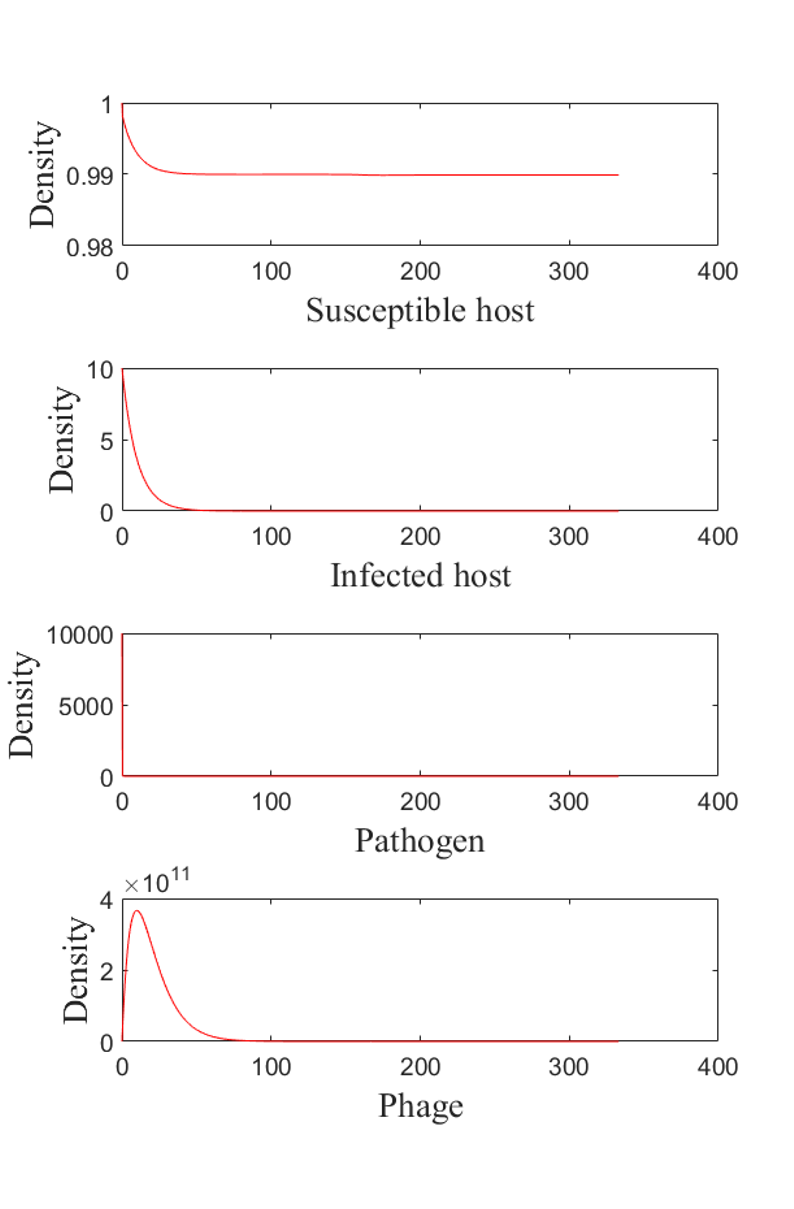

Supplement: Supplementary file 4 — Figure S3 Time dynamics of pathogen, susceptible, infected host and bacteriophage dynamics (S-I-P-F) when the phage transmission and release rate are high (βF = 10− 3 and ΛF = 100). X-axis shows time in days and y-axis the population density. The pathogen population decreases close to extinction almost immediately, while there is a delay of approximately 47 days for the elimination of the disease and more than 50 days for decrease of the phage population close to extinction. The parameter values used are shown in Table 1. (TIF 2818 kb) [file 12976_2018_79_MOESM4_ESM.tif]
