# Supplementary material for: Outside-host phage therapy as a biological control against environmental infectious diseases
Source: Theor Biol Med Model. 2018 Jun 8;15:7. doi: 10.1186/s12976-018-0079-8 (PMC5992827; doi:10.1186/s12976-018-0079-8)
Supplement: Supplementary file 2 — Equilibrium population densities and the Jacobian matrices for phage therapy model (1)–(6). Local stability analysis of the P-S-I submodel at S=I = 0 in the absence of recovery. (DOCX 30 kb) [file 12976_2018_79_MOESM2_ESM.docx]

## Phage therapy as a biological control against environmental infectious diseases

Ilona Merikanto^1#^, Jouni T. Laakso^1,2^, Veijo Kaitala^1^

**Additional file 2. Equilibrium population densities and the Jacobian matrices for phage therapy model (1)-(6)**

Local stability properties of the equilibria were determined by studying numerically the eigenvalues of the Jacobian matrices obtained by linearizing the equations at the equilibrium.

The Jacobian matrix of the model (1)-(6) is obtained by linearizing it at the equilibrium:

$$J=\left[ \begin{matrix} \begin{matrix} \begin{matrix} r_{S}-2r_{S}\bar{S}-\beta\overline{P}-\beta_{B}\overline{B}-\mu_{SI} & \delta& \delta\\ \beta\overline{P} & -\left( \alpha+\mu_{SI}+\delta\right) & 0 \\ \beta_{B}\overline{B} & 0 & -\left( \alpha+\mu_{SI}+\delta\right) \end{matrix} \\ \begin{matrix} 0 & \Lambda\alpha& 0 \\ 0 & 0 & \Lambda_{B}\alpha\\ 0 & 0 & 0 \end{matrix} \end{matrix} & \ldots\end{matrix} \right.$$

$$\left. \begin{matrix} \ldots& \begin{matrix} \begin{matrix} -\beta\bar{S} & -\beta_{B}\bar{S} & 0 \\ \beta\bar{S} & 0 & 0 \\ 0 & \beta_{B}\bar{S} & 0 \end{matrix} \\ \begin{matrix} r_{P}\left( 1-\frac{2\bar{P}+\bar{B}}{K} \right)-\mu_{P}-\beta_{F}\overline{F} & -r_{P}\frac{\bar{P}}{K} & {-\beta}_{F}\overline{P} \\ -\frac{r_{B}\bar{B}}{K} & r_{B}\left( 1-\frac{\bar{P}+2\bar{B}}{K} \right)-\mu_{B} & 0 \\ \Lambda_{F}\beta_{F}\overline{F} & 0 & 0 \end{matrix} \end{matrix} \end{matrix} \right]$$

The Jacobian matrices of subsystems, e.g., *B*=0 or *F*=0, are obtained as a corresponding submatrix of the general Jacobian matrix *J*.

The equilibrium densities of the general model (1)-(6) values are given as

$$\overline{P}=\frac{\mu_{F}}{\Lambda_{F}\beta_{F}}$$

$\overline{B_{1}}=0$ or

$$\overline{B_{2}}=-\frac{A_{2}}{A_{1}}$$

where

$$A_{1}=-\frac{\Lambda_{B}\alpha{\beta_{B}}^{2}}{r_{S}\left( \alpha+\mu_{SI}+\delta\right)}-\frac{\delta\Lambda_{B}\alpha{\beta_{B}}^{2}}{r_{S}{(\alpha+\mu_{SI}+\delta)}^{2}}-{(r}_{B}/K)$$

$$A_{2}={\frac{\Lambda_{B}\alpha\beta_{B}}{r_{S}\left( \alpha+\mu_{SI}+\delta\right)}\left( r_{S}-\beta\overline{P}-\mu_{SI}-\frac{\delta\beta\overline{P}}{(\alpha+\mu_{SI}+\delta)} \right)+r}_{B}\left[ 1-\frac{\bar{P}}{K} \right]-\mu_{B}$$

and

$$\overline{S}=\frac{(r_{S}-\beta\overline{P}-\beta_{B}\overline{B}-\mu_{SI})}{r_{S}}+\frac{\delta\beta\overline{P}}{r_{S}\left( \alpha+\mu_{SI}+\delta\right)}+\frac{{\delta\beta}_{B}\overline{B}}{r_{S}(\alpha+\mu_{SI}+\delta)}$$

$$\overline{I}=\frac{\beta\overline{S}\overline{P}}{(\alpha+\mu_{SI}+\delta)}$$

$$\overline{I}_{B}=\frac{\beta_{B}\overline{S}\overline{B}}{(\alpha+\mu_{SI}+\delta)}$$

and

$$F=\left[ \Lambda\alpha I+r_{P}(1-\frac{\bar{P}+\bar{B}}{K})\bar{P}-\mu_{P}\bar{P} \right]/\beta_{F}\bar{P}$$

In the absence of the phage (F=0, Fig. 3) the equilibrium densities of the model are obtained as

$$\left[ \begin{matrix} \bar{S} \\ \bar{P} \\ \bar{B} \end{matrix} \right]=\left[ \begin{matrix} 1 & \frac{\beta}{r_{S}}(1-\frac{\delta}{\left( \alpha+\mu_{SI}+\delta\right)}) & \frac{\beta_{B}}{r_{S}}(1-\frac{\delta}{\left( \alpha+\mu_{SI}+\delta\right)}) \\ -\frac{\Lambda\alpha\beta}{\alpha+\mu_{SI}+\delta} & \frac{r_{P}}{K} & \frac{r_{P}}{K} \\ -\frac{\Lambda_{B}\alpha_{B}\beta_{B}}{\alpha_{B}+\mu_{SI}+\delta} & \frac{r_{B}}{K} & \frac{r_{B}}{K} \end{matrix} \right]^{-1}\left[ \begin{matrix} 1-\mu_{SI}/r_{S} \\ r_{P}-\mu_{P} \\ r_{B}-\mu_{B} \end{matrix} \right]$$

and *I* and *I_B_* are obtained from equations (2) and (3). If the host S becomes extinct and the growth and mortality rates are equal for the bacteria (Fig. 3) then the equilibria levels are not uniquely defined.

**Local stability analysis of the P-S-I submodel at S=I=0 in the absence of recovery**

Assume that the host is extinct: *S*=*I*=0. Then the equilibrium level of the pathogen is ${\bar{P}=(r}_{P}-\mu_{P})K/r_{P}$.

The Jacobian matrix becomes

$$\left\lceil\begin{matrix} r_{S}-\beta\bar{P}-\mu_{SI} & 0 & 0 \\ \beta\bar{P} & -\left( \alpha+\mu_{SI} \right) & 0 \\ 0 & \Lambda\alpha& {-(r}_{P}-\mu_{P}) \end{matrix} \right\rceil$$

The eigenvalue of the $\lambda=r_{S}-\beta\bar{P}-\mu_{SI}$defines the local stability properties of the equilibrium S=I=0 since the two other eigenvalues are negative. Thus, if $\lambda=r_{S}-\beta\bar{P}-\mu_{SI}<0$ then the extinction of the host is locally stable, and if $\lambda>0$ then it is unstable allowing recovery.
